# Global Dynamics of Yeast Hsp90 Middle and C-Terminal Dimer Studied by Advanced Sampling Simulations

**DOI:** 10.3389/fmolb.2019.00093

**Published:** 2019-09-27

**Authors:** Florian Kandzia, Katja Ostermeir, Martin Zacharias

**Affiliations:** Physics Department T38, Technical University of Munich, Garching, Germany

**Keywords:** Hsp90 conformational dynamics, biasing potential REMD, advanced sampling simulations, sampling global dynamics, multi-scale dynamics sampling, Hsp90 chaperone function

## Abstract

The Hsp90 protein complex is one of the most abundant molecular chaperone proteins that assists in folding of a variety of client proteins. During its functional cycle it undergoes large domain rearrangements coupled to the hydrolysis of ATP and association or dissociation of domain interfaces. In order to better understand the domain dynamics comparative Molecular Dynamics (MD) simulations of a sub-structure of Hsp90, the dimer formed by the middle (M) and C-terminal domain (C), were performed. Since this MC dimer lacks the ATP-binding N-domain it allows studying global motions decoupled from ATP binding and hydrolysis. Conventional (c)MD simulations starting from several different closed and open conformations resulted in only limited sampling of global motions. However, the application of a Hamiltonian Replica exchange (H-REMD) method based on the addition of a biasing potential extracted from a coarse-grained elastic network description of the system allowed much broader sampling of domain motions than the cMD simulations. With this multiscale approach it was possible to extract the main directions of global motions and to obtain insight into the molecular mechanism of the global structural transitions of the MC dimer.

## Introduction

The 90 kDa heat-shock protein (Hsp90) is an essential molecular chaperon protein that plays a vital role in the folding process of several client proteins (Hunter and Poon, 1997; Mayer and Bukau, 1999; MacLean and Picard, 2003; Pratt and Toft, 2003; Prodromou and Pearl, 2003; Pratt et al., 2004). It is found in bacteria as well as eukaryotes and is essential for cell viability and plays a pivotal role in many signaling and regulation pathways (Echeverría et al., 2011). In its active conformation it forms a homodimer and its chaperone activity depends on ATP binding and hydrolysis. During its work cycle Hsp90 and its homologs (e.g., HtpG, Grp94, Trap1) can adopt different global conformations covering a range of tightly bound closed structures up to widely open conformations (Harris et al., 2004; Ali et al., 2006; Shiau et al., 2006; Dollins et al., 2007; Lavery et al., 2014; Verba et al., 2016). For example, the crystal structure of yeast Hsp90 bound to a non-hydrolysable ATP analog (AMPPNP) indicates a closed homodimer with domain contacts between the N-terminal (N)-domain and C-terminal (C)-domain of each monomer (the N- and C-domains in each monomer are connected by a middle (M)-domain) (Ali et al., 2006). A similar structure was obtained for a complex of yeast Hsp90 and a kinase client protein (Verba et al., 2016). Furthermore, structures of a paralog, Grp94, from the mammalian endoplasmic reticulum (ER) (Dollins et al., 2007) and of HtpG (bacterial homolog) (Shiau et al., 2006) are known. The Grp94 adopts a slightly more open structure compared to yeast Hsp90 and the HtpG homolog is dramatically more open. Studies employing small-angle X-ray scattering (SAXS) indicate that the Hsp90 conformation depends significantly on the bound nucleotide. Using a large number of fluorescence donor and acceptor pairs a recent single molecule FRET (fluorescence resonance energy transfer) study was used to obtain ensembles of Hsp90 conformations in the apo state and in the presence of ADP, AMPPNP (Hellenkamp et al., 2016). These studies confirmed the known Hsp90 structure in the presence of AMPPNP and indicated a more open structure and reorientation of the N-domain compared to the closed conformation when ADP is bound or for the APO state (Hellenkamp et al., 2016). Based on restraint MD-simulations atomistic structural models compatible with the sFRET data for the open yeast Hsp90 state in the presence of ADP have been obtained (Hellenkamp et al., 2016). However, the mechanism how ATP hydrolysis (or loss of a bound nucleotide) can trigger global domain rearrangements is still not clear. The observation that a loss of the interaction between the N-domains results in a global opening indicates that the closed (ATP-bound form) corresponds to a structure under global stress (the unfavorable global deformation away from the open form is stabilized by the N-domain binding). Hence, a removal of the N-domain interaction should result in an opening of the Hsp90 structure. Indeed, the crystal structure of a truncated form of Hsp90 (without the N-domains) shows still the same C-domain dimerization contacts compared to the full Hsp90 structure but an increased distance between the M-domains toward a more open global conformation (**Figure 2D**). However, the degree of opening is significantly smaller compared to the “open” ADP-bound structure based on the sFRET data (**Figure 2E**). The origin of this discrepancy could be crystal contacts that may stabilize only one type of global conformation among other global arrangements that are accessible in free solution.

In order to elucidate the global conformational flexibility of the yeast Hsp90 MC dimer (Hsp90 without N-domain) in solution we performed a series of comparative Molecular Dynamics (MD) simulations starting from different initial conformations. The initial structures corresponded to the known crystal structure of the yeast Hsp90 MC dimer, the crystal structure of the closed full Hsp90 (AMPPNP-bound), a single molecule FRET derived start structure and an arrangement based on a bacterial homolog (in a wide open geometry) (Ali et al., 2006; Shiau et al., 2006; Hellenkamp et al., 2016). The transition between open (ADP-bound) and closed (ATP-bound) states of the full length Hsp90 occurs on the μs to ms time scale (Hellenkamp et al., 2016). However, it is expected that in case of the truncation of the N-domain (that is the primary interaction partner to stabilize the closed form) global transitions and conformational relaxations in the MC dimer occur much faster compared to the full structure and allow to identify the associated molecular details of the global domain motions.

In order to further enhance the sampling of global motions we also performed Hamiltonian replica exchange (H-REMD) simulations coupled with an elastic network model (ENM) description of the MC dimer. A low resolution representation of protein dynamics can be obtained using coarse-grained elastic network models (ENM) to extract directions of global mobility (Bahar and Rader, 2005; Bastolla, 2014). Recently, we have developed a H-REMD approach that uses information from an ENM analysis and combines it with atomistic MD simulations in explicit solvent (Ostermeir and Zacharias, 2014).

The approach forms an effective multi-scale methodology in which directions of large scale global conformational transitions (extracted from a low resolution technique) can guide and enhanced the high-resolution atomistic sampling of the multi-domain structure.

Indeed, the unrestrained MD simulations starting from different initial conformations of the Hsp90 MC dimer with globally different initial domain arrangements sampled only conformations relatively close to the starting structures on a time scale of 200 ns. On the other hand, the ENM-coupled REMD methodology sampled a much wider range of domain arrangements including relatively close but also more open Hsp90 MC dimer structures.

The results indicate, firstly, that the ENM-REMD method is an efficient multi-scale enhanced sampling technique offering improved sampling compared to regular MD simulations. Secondly, our simulations demonstrate that in the absence of the N-domains the Hsp90 dimer can adopt a variety of closed and open domain arrangements that might be of functional importance for chaperone function. One function of the N-domain might be to limit these possible states by N-domain dimerization that is controlled by the bound ATP or ADP nucleotide.

## Materials and Methods

Four model structures of the MC dimer of yeast Hsp90 were build corresponding to published structures of Hsp90 and its homologs [pdb2cg9 (Ali et al., 2006), pdb2cge (Ali et al., 2006), pdb2ioq (Shiau et al., 2006), and the mean open structure as determined by Hellenkamp et al. (2016)]. Since the 2cge structure corresponds to a Hsp90 MC domain dimer the published structure served directly as start structure representing the 2cge-model. For the models based on the closed 2cg9 full length structure and the full length mean open structure the N-terminal domain segments up to the start of the middle domain (residue 1–236) were removed forming the 2cg9- and sFRET-models, respectively. A starting model based on the bacterial homolog 2ioq was generated by superimposing the M- and C-domains from the yeast 2cge structure onto the corresponding conserved elements of the 2ioq homolog using Pymol (Schrodinger, 2015), resulting in a start structure with an overall Cα-Rmsd value of 3.4 Å relative to the corresponding elements in the 2ioq structure. Solvation of the structures was performed in octahedral boxes with explicit water molecules (TIP3P) (Jorgensen et al., 1983) and neutralized with chloride and sodium ions up to an ion concentration of 0.1 M using the leap module and employing the parm14SB force field (Maier et al., 2015) of the Amber14 package. All unrestrained simulations were performed using the pmemd.cuda code of the Amber14 package (Case et al., 2014). The start structures were energy minimized using steepest descent and conjugated gradient methods (5,000 steps), and slowly heated up to 300 K in 500 ps NVT run using Langevin dynamics, while restraining all heavy atoms with respect to the start structure. During another 150 ps the positional restraints were reduced in a step-wise manner, allowing the system to relax. The systems were further equilibrated for 200 ps at constant pressure using a Berendsen barostat followed by a 200 ns data gathering period. During all simulations the Particle Mesh Ewald (PME) method was used to calculate long range electrostatic interactions (Darden et al., 1993) with a real space cutoff radius of 9 Å. The Shake algorithm was used to constrain bonds involving hydrogen atoms (Ryckaert et al., 1977), which allowed employing a time step of 2 fs.

For the ENM-coupled H-REMD simulations we followed a published protocol. The H-REMD simulations involve a biasing potential that acts between domain centroids of the multi domain protein. In case of the Hsp90 MC dimer 4 centroids representing the centers (based on the protein Cα atoms) of the four domains were used. The protein conformational fluctuations are calculated by means of an elastic network model for the protein Cα atoms based on Hinsen (Hinsen, 1998) and following the protocol described in Ostermeir and Zacharias (Ostermeir and Zacharias, 2014). The first 50 normal modes were excited by a thermal energy of RT (R: gas constant and T, temperature = 300 K) that reflects the possible distance fluctuations between the domains from the ENM analysis. In the ENM-coupled REMD approach the biasing potential is generated to specifically enhance structural changes in the REMD simulations compatible with the fluctuation obtained from the ENM and to destabilize the domain arrangement along the centroid distances d_ij_ (i,j are centroid labels).

$$\begin{array}{rc} V\left(d_{ij}\right)\;=k{\left({\left[d_{ij}-d_{ij0}\right]}^{2}-\Delta d_{ij}^{2}\right)}^{2},\;if\;|d_{ij}-d_{ij0}|\leq \Delta d_{ij}\\ \;V\left(d_{ij}\right) & =0,\;otherwise \end{array}$$

In the H-REMD one reference replica was run under the control of the original force field whereas the centroid-centroid distance dependent biasing potentials were added with increasing amplitudes in each of the 11 replicas (total replica number: 12). A replica exchange was attempted every 2 ps. The magnitudes and the width of the biasing potentials in the replicas were adjusted during the simulations with a starting biasing level of 2.25 kcal/mol (corresponding to ~4 RT) between replicas at the beginning. Centroid-centroid distances were updated every 0.2 ns from the running average of the last 0.4 ns. The BP-amplitude was also adjusted every 0.2 ns to optimize the acceptance rate of replica exchanges. If the acceptance probability for exchanges between neighbors decreased to <20% (or surpasses 60%) in any of the replicas the BPs were lowered (or increased) by 10%. After the first 5 ns of the H-REMD the biasing levels stabilized to ~0.45 RT between replicas and remained constant for the rest of the simulation within a standard deviation of ± 0.12 RT. The REMD simulations were extended for 25 ns. More details on the ENM coupled REMD methodology are given in reference 17. Simulation results were analyzed by means of the cpptraj module of Amber14 (Case et al., 2014).

## Results and Discussion

**Figure 1:**
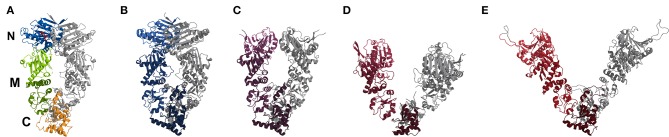
Comparison of crystal structures (cartoon representation) of yeast Hsp90 with bound AMPNPP (**A**, pdb2cg9), a Hsp90-Cdc37-Cdk4 complex bound with ATP (**B**, pdb5fwl), a mitochondrial homolog with bound ADP (**C**, pdb2o1v), a bacterial homolog with bound ADP (**D**, pdb2ioq) and an “open” ADP-bound structure based on sFRET data (**E**, Hellenkamp et al., 2016). In **(A)** the location of the N-, M-, and C-domains is indicated and color-coded for one monomer, the second monomer is in gray. In case of **(B–E)** one monomer is colored from light (N) to dark (C), while the other is in gray for visibility. The colors range from blueish (closed conformation) to reddish (open conformation).

**Figure 2:**
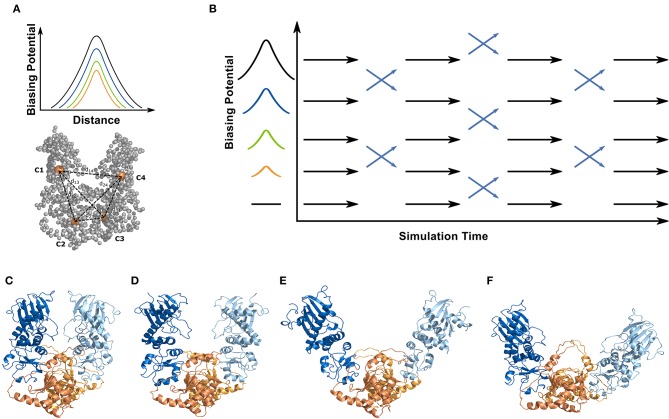
Simulation systems and setup for the ENM coupled REMD simulations. All simulations were performed on a truncated yeast Hsp90 consisting of a dimer of the M- and C-domains (without N-domain). For the ENM-coupled REMD the center-of-mass of the M- and C-domains (orange spheres, C1-C4) served as the 4 centers to design a biasing potential in the replica runs (illustrated in **A**). The biasing potential acts along the distances between the 4 centers (six distances) indicated as dashed lines. The magnitude of the biasing potential increases along the replicas (indicated in **B**) and the width is obtained from the corresponding distance fluctuation derived from the ENM calculations of the protein. The ENM coupled REMD is schematically illustrated in **(B)**. The differences in global opening of the start structures used in the 2cg9 **(C)**, the 2cge **(D)**, the sFRET **(E)**, and the 2ioq **(F)** simulations is also indicated. The domains are color coded in the cartoon representation in **(C–F)** (M-domain of first monomer in blue/C-domain in orange; M-domain of second monomer in light blue/C-domain in light orange).

**Figure 3:**
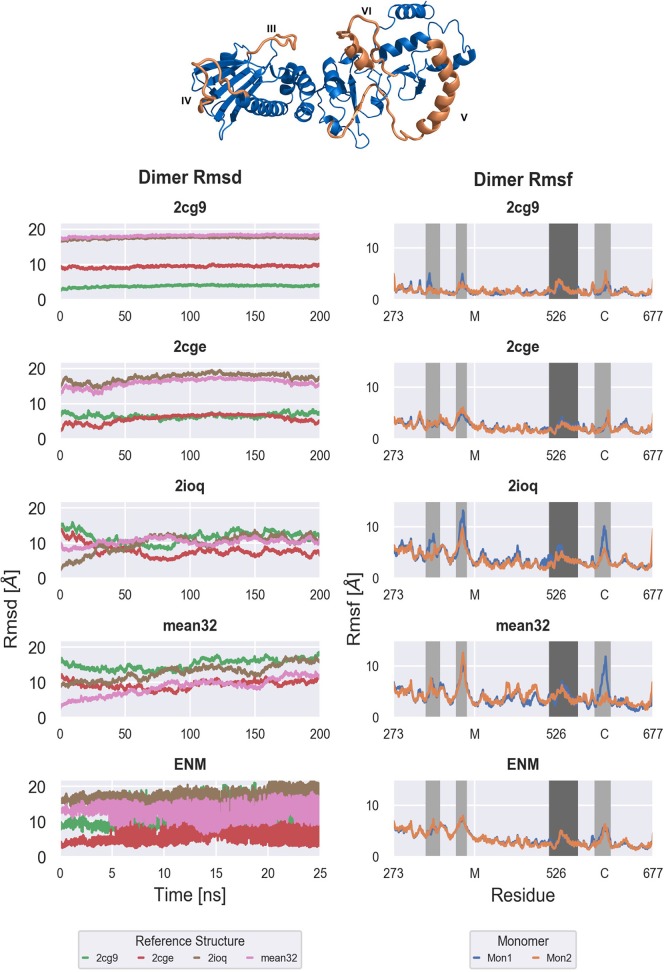
Backbone Rmsd of the full M-C dimer with respect to the generated models **(Left)** and per residue Rmsf of both monomers of the M-C dimer **(Right)**. From top to bottom: Free MD simulations ranging from tightly closed (2cg9) over semi closed (2cge) to widely open (2ioq, sFRET) conformations and ENM-coupled REMD simulations based on the semi closed (2cge) conformation. Right: Important regions are highlighted in dark [M-C linker (V)] and light gray [amphipatic loop (III), M loop (IV), C helix 2 (VI)]. The structure of one M-C monomer is shown as cartoon in blue with the important regions highlighted in orange and annotated.

The Hsp90 chaperone homodimer undergoes dramatic global conformational changes during its working cycle that are accompanied by binding of the N-domains (if ATP is bound) or dissociation of the N-domains (ADP-bound and apo states). During its functional cycle the C-domains stay always in a bound state (Figure 1). How ATP hydrolysis triggers N-domain dissociation and the subsequent global opening is not fully understood. If N-domain dissociation triggers global opening removal of the N-domains should also allow large scale global motions in the truncated Hsp90 MC dimer. Comparative MD simulations starting from closed intermediate open and fully open conformations were used to investigate the global mobility of the MC dimer. As described in the Methods section the start structures corresponded to the crystal structure of the Hsp90 MC dimer (pdb2cge) that can be considered as semi-open conformation (Figure 2D) compared to the structure found in the closed state (pdb2cg9) that formed another start conformation (Figure 2D). More open states are based on the recent single molecule FRET analysis in the presence of ADP (Hellenkamp et al., 2016), termed sFRET-conformation (Figure 2E) and another open conformation based on the bacterial homolog (pdb2ioq) (Figure 2F). For each unrestrained MD simulation, we recorded the deviation with respect to each of the four reference structures (Figure 3).

Starting from the closed conformation (2cg9-start) results in almost constant root-mean square deviation (RMSD of the backbone) during the simulation time (Figure 3). On the time scale of the simulation no tendency for global opening is observed (the RMSD with respect to the more open start structures remains constant). Similarly, in case of starting from the 2cge reference only small shifts in the RMSD is observed although the fluctuations in the RMSD are clearly larger compared to the simulations based on the 2cg9-start. Again little tendency toward larger global changes (closer approach of the more open conformations) is observed. To the contrary, the RMSD with respect to the more closed 2cg9-model is slightly decreasing over the simulation time. Note, that in both the 2cge as well as the 2cg9 conformations the M-domains are in close contact with the C-domains mediated by two large loop regions that are part of the C-domains (Figure 1) and that may transiently stabilize the global arrangement.

For the simulations starting from the open conformations, 2ioq and sFRET(ADP), the Rmsd decreases w.r.t. the semi open conformation of 2cge, while it increases w.r.t. the open conformations of 2ioq and sFRET. This is caused by a motion with the overall slight tendency of closing but not exactly reaching the 2cge structure (Rmsd > 8–10 Å). Note, that in the open form the contact between the loop region of the C-domain and the M-domain is largely missing that may allow a great global mobility.

In contrast to the unrestrained MD simulations (starting from the 2cge-structure) the reference replica of the ENM coupled REMD simulations showed much larger changes in the Rmsd over time (and on shorter time scale, Figure 3) with respect to all reference structures. Note, that the rapid changes observed in the [Figure 3 (panel labelled ENM)] are due to the exchanges between conformations in neighboring replicas indicating that a great variety of different conformations is sampled in this scheme. During the ENM coupled REMD distance-dependent biasing potentials are derived from the ENM analysis that act between the domain segments (C1,.,C5; illustrated in Figure 2). These potentials promote motions in the soft collective directions of the system (in the replicas) and result in enhanced domain motion sampling (without providing any preset reaction coordinate for the global motions of the domains such as domain angles or dihedrals).

It is of interest to identify the origin of conformational fluctuations. One possible source are local conformational fluctuations within each MC monomer. One possibility to analyze the flexibility of each monomer is to investigate the mean fluctuations (RMSF) along each monomer (Figure 3) and the buried surface area along the sequence (Supplementary Figure 1). The pattern of conformational fluctuations looks qualitatively similar in all simulations with 4 regions indicating enhanced local mobility compared to the mean of the structures (Figure 3). In each of the simulations the pattern is similar for both monomers (compare orange and blue lines in Figure 3). In general, the magnitude of fluctuations is smaller for the 2cg9- and 2cge-simulations compared to the simulations starting from the open Hsp90 MC dimers or the ENM coupled REMD simulations. The more flexible regions are highlighted in light and dark gray. In the M domain an amphiphatic (III) and a flexible loop (IV) create strong interfaces with the opposite monomer that stabilize the closed conformations (Figure 3). In the simulations starting from the open conformations these loops are highly dynamic (Figure 3) and are much less mobile in case of the simulations starting from more closed MC dimers. It is possible that the interaction of the loop IV region with the M-domain may initiate closing movements in Hsp90. Other regions that show significant differences in mobility are located more near to the N-terminus (regions III, IV, Figure 3). Also, the M-domain indicates more fluctuations in the 2ioq, sFRET, and the ENM-coupled REMD simulations compared to the 2cg9 and 2cge MC dimer simulations. The C-terminal part of the dimer appears to be more rigid and inherits only a low mobility in all simulations and for all starting conformations.

**Figure 4:**
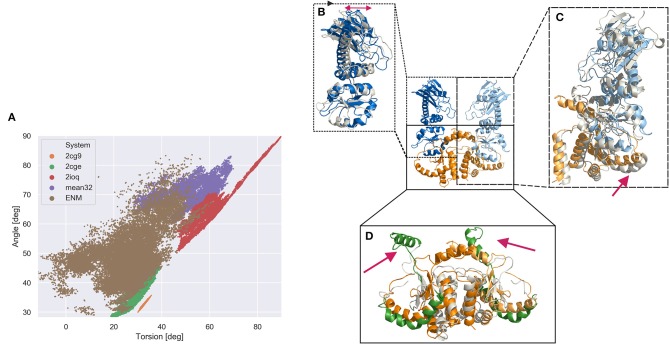
Global domain sampling recorded during MD simulations and ENM coupled REMD simulations in terms of a Hsp90 MC dimer angle and dihedral torsion (defined using the center coordinates c1, c2, c3, c4 shown in Figure 2A; angle formed by c1, (c2, c3), c4; dihedral torsion formed by c1, c2, c3, c4). **(A)** Each data point represents a sampled domain arrangement. (brown dots) Reference replica of the ENM coupled REMD (25 ns), (green dots) unrestrained MD simulation starting from 2cge structure (200 ns), (orange dots) simulation starting from MC dimer extracted from 2cg9 structure (200 ns), (purple dots) MD simulation starting from sFRET derived open Hsp90 conformation (200 ns), (red dots) sampling obtained from MD simulation starting from arrangement in bacterial homolog pdb2ioq (200 ns). **(B–D)** characteristic structural domain changes observed during the ENM coupled REMD simulation indicated by a snapshot superimposed on the start structure. **(B)** Within the M-domain a hinge like motion of the upper part vs. lower part of the M-domain is observed (indicated by a red double arrow). **(C)** The change of one M-domain relative to the C-domain is associated to a movement of the connecting helix (red arrow) **(D)** within the C-domains the C helix 2 (VI) segments (see Figure 2) undergo large scale motions (highlighted in green and indicated as red arrows) relative to the position in the start structure.

The magnitude of the local RMSF of residues along each monomer cannot explain the large RMSD shifts and changes observed especially in the ENM coupled REMD simulations. As a next step we analyzed the global conformational fluctuations observed in the complete MC dimer. The global opening angle and the torsional dihedral angles described by the four domains (illustrated in Figure 2) might be considered as most useful and intuitive variables to illustrate and analyze the global domain motion. With respect to these variables the 2cg9 simulation indicates the least global mobility on the present simulation time scale. Apparently, it is locked in a locally stable arrangement that allows only limited local as well as global motions (on the present 200 ns time scale). More global mobility is observed for the 2cge case and even broader distributions are found for the simulations starting from the sFRET and from the 2ioq start structures (Figure 4). However, only the latter unrestrained simulations show some overlap of sampled global variables but there is no overlap between states sampled in the 2cge and 2cg9 simulations and those starting from the open model structures. Apparently, there are barriers between states or a low diffusivity on the global energy landscape that prevents the observation of global transitions in the unrestrained MD simulations on the 200 ns time scale.

However, a much broader covered range of sampled global opening angle and global dihedral torsion angle of the domains is sampled in the ENM coupled REMD simulations. The sampling overlaps very well with the states sampled in the 2cge simulation (it also started from the 2cge MC dimer structure), and also at least partially overlap with the sampling seen in the simulations that started from sFRET-derived start arrangement and the 2ioq-based simulations but covers also many more arrangements (Figure 4). Since the ENM coupled REMD technique involves an active driving force along the global variables (in the present case the domain distances and in the higher replicas of the REMD) it can more easily overcome small energy barriers and slows down global motions much less due to low domain diffusivity. The low diffusivity can be caused by many transiently stable interactions of equivalent stability but that need to be continuously disrupted and re-established during diffusive global motion. The broad sampling observed in the reference replica of the ENM coupled REMD indicates that large regions in the space of the two global variables are in principle accessible (are of equivalent free energy) that correspond to mostly open conformational states.

Interestingly, even in the REMD simulations (but similarly also in the 2cge based unrestrained MD-simulations) no conformations that closely approaches the 2cg9 structure were sampled. It is possible that the transition to the closed 2cg9 form involves a significant energy barrier and simultaneous rearrangement of interface residues between the C-domain loop segment and the M-domain that was not sampled during the relatively short ENM coupled REMD simulations. This assumption is further supported by the relatively small and very confined region in the two global variables that is sampled when starting from the 2cg9 conformation. Such confined sampling indicates the existence of energy barriers that prevent dissociation processes to trigger global opening motions. Indeed, a comparison of the 2cg9 and 2cge start structures indicates several additional contacts in the 2cg9 case. This includes the disruption of these contacts may cause the energy barrier. Vice versa simulations starting from the 2cge (or other more open forms) face a penalty to form the correct contacts between M-domains before reaching the most closed 2cg9 state. Future ENM coupled REMD simulations or other advanced sampling techniques starting from the 2cg9 structure might be useful to investigate such putative energy barriers.

The simulation results can also be used to structurally characterize local changes that might be coupled to the observed global domain motions (Figure 4). The sampled open conformations of the MC dimer in the ENM-REMD indicate large local conformational changes especially in the helix connecting the M and C-domain (region V in Figure 2) that partially unfolds during domain opening motions (Figure 4). In addition, large motions of the C-helix 2 (region VI in Figure 2) are observed in the sampled states that represent more open domain arrangements in the REMD run (Figure 4). It is indeed this C-helix 2 region IV that mediates contacts between C-domains and between C-domains and the M-domains in the closed form (see Figure 2C). The interaction is partially broken in the 2cge form and largely lost in the open forms (compared Figures 2C,F) as well as in the ENM-coupled H-REMD simulations.

## Conclusions

Depending on the nucleotide-bound state Hsp90 can adopt different global domain arrangements. The stability of the domain arrangements is controlled by the binding of nucleotides to the N-domain. In the present simulations also different locally stable domain arrangements of the Hsp90 MC dimer (lacking the N-domain) were observed that do not undergo transitions in standard MD-simulations on the time scale of 200 ns. This indicates that not only N-domain interactions but also interactions of the other domains influence the global Hsp90 structure. The ENM-REMD technique that combines an atomistic description of the system with global mobile directions observed in a coarse-grained ENM was shown to more effectively sample the globally accessible space for the Hsp90 MC dimer. Future applications of the technique to the Hsp90 molecule including the N-domains could be useful to elucidate global motions in the full Hsp90 molecule.

## Data Availability Statement

All datasets generated for this study are included in the manuscript/Supplementary Files.

## Author Contributions

MZ designed and supervised research. FK performed MD simulations and analyzed data. KO performed replica exchange simulations. All authors contributed to writing of the manuscript.

### Conflict of Interest

The authors declare that the research was conducted in the absence of any commercial or financial relationships that could be construed as a potential conflict of interest.
