# Supplementary material for: Global Dynamics of Yeast Hsp90 Middle and C-Terminal Dimer Studied by Advanced Sampling Simulations
Source: Front Mol Biosci. 2019 Sep 27;6:93. doi: 10.3389/fmolb.2019.00093 (PMC6798034; doi:10.3389/fmolb.2019.00093)
Supplement: Supplementary file 1 [file Data_Sheet_1.PDF]

Supplementary Material

Global dynamics of yeast Hsp90 middle and C-terminal dimer studied by advanced sampling simulations

Florian Kandzia, Katja Ostermeir and Martin Zacharias*

Technical University of Munich, Physics Department T38, James-Franck-Str. 1, 85748 Garching, Germany

*corresponding author
e-mail: martin.zacharias@ph.tum.de

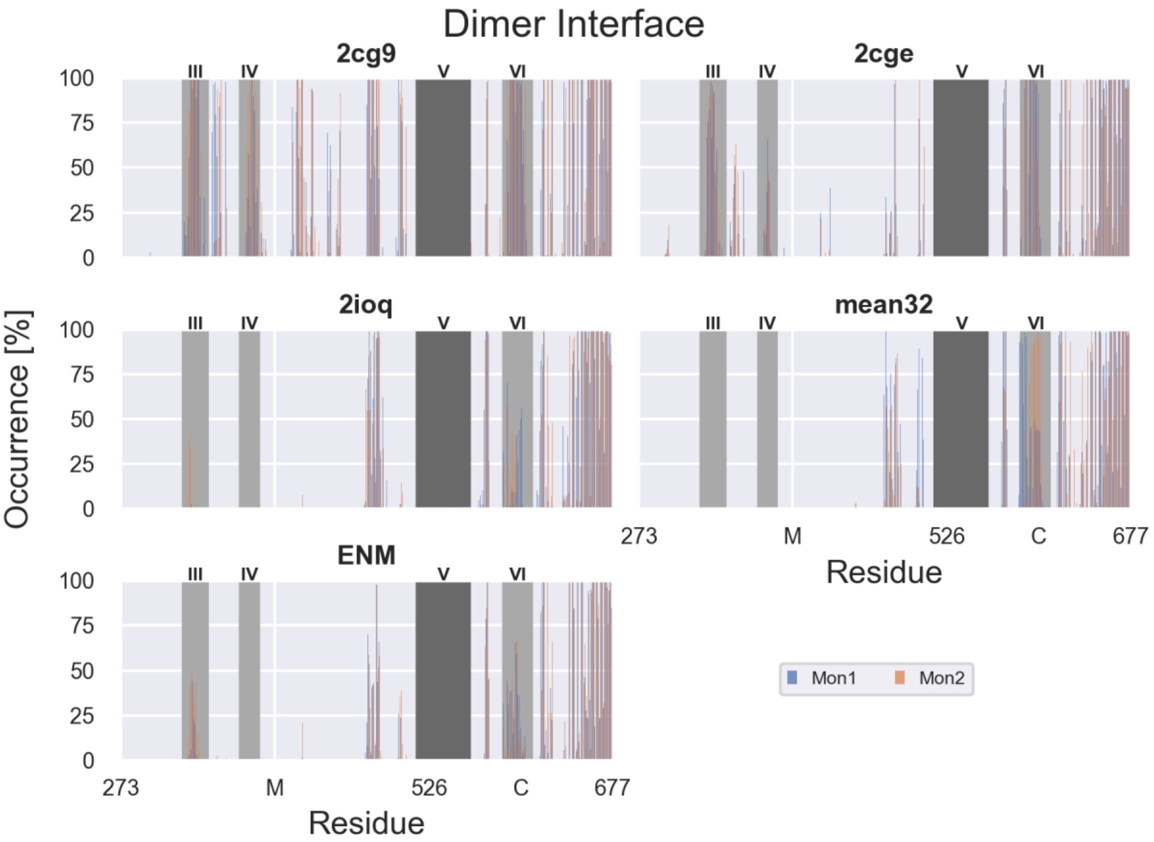

Supplementary Material Figure 1: Inter-monomer interface calculated based on buried surface area (Bsa) for the free simulations and the ENM simulation. Important regions are highlighted in dark (M-C linker (V)) and light grey (amphipatic loop (III), M loop (IV), C helix 2 (VI)).
